# Expression Analysis of Hairpin RNA Carrying* Sugarcane mosaic virus* (SCMV) Derived Sequences and Transgenic Resistance Development in a Model Rice Plant

**DOI:** 10.1155/2017/1646140

**Published:** 2017-02-01

**Authors:** Sehrish Akbar, Muhammad Tahir, Ming-Bo Wang, Qing Liu

**Affiliations:** ^1^Atta-ur-Rahman School of Applied Biosciences (ASAB), National University of Sciences and Technology (NUST), Islamabad 44000, Pakistan; ^2^Commonwealth Scientific Industrial and Research Organization, Agriculture, GPO Box 1600, Canberra, ACT 2601, Australia

## Abstract

Developing transgenic resistance in monocotyledonous crops against pathogens remains a challenging area of research.* Sugarcane mosaic virus* (SCMV) is a serious pathogen of many monocotyledonous crops including sugarcane. The objective of present study was to analyze transgenic expression of hairpin RNA (hpRNA), targeting simultaneously* CP* (Coat Protein) and* Hc-Pro* (helper component-proteinase) genes of SCMV, in a model rice plant. Conserved nucleotide sequences, exclusive for DAG (Aspartic acid-Alanine-Glycine) and KITC (Lycine-Isoleucine-Threonine-Cysteine) motifs, derived from SCMV* CP* and* Hc-Pro* genes, respectively, were fused together and assembled into the hpRNA cassette under maize ubiquitin promoter to form Ubi-hpCP:Hc-Pro construct. The same CP:Hc-Pro sequence was fused with the *β*-glucuronidase gene (GUS) at the 3′ end under CaMV 35S promoter to develop 35S-GUS:CP:Hc-Pro served as a target reporter gene construct. When delivered into rice callus tissues by particle bombardment, the Ubi-hpCP:Hc-Pro construct induced strong silencing of 35S-GUS:CP:Hc-Pro. Transgenic rice plants, containing Ubi-hpCP:Hc-Pro construct, expressed high level of 21–24 nt small interfering RNAs, which induced specific suppression against GUS:CP:Hc-Pro delivered by particle bombardment and conferred strong resistance to mechanically inoculated SCMV. It is concluded that fusion hpRNA approach is an affordable method for developing resistance against SCMV in model rice plant and it could confer SCMV resistance when transformed into sugarcane.

## 1. Introduction

*Sugarcane mosaic virus* (SCMV), belonging to genus* Potyvirus*, family Potyviridae, is a serious pathogen of many monocotyledonous crops including sugarcane. In Pakistan, approximately 10–32% losses has been estimated in sugarcane yield which results in 6–10% decline in sugar production [1]. Infected plants showed mosaic pattern and are characterized by typical pinwheel shaped inclusion bodies in cell cytoplasm. The 10 kb single stranded RNA genome of SCMV encodes a single polypeptide which is cleaved either co- or post translationally into ten mature proteins (*P1, HC-Pro, P3, 6K1, CI, 6K2, VPg, NIa-Pro, NIb,* and* CP*) [2, 3]. C-terminus of the polyprotein encodes the coat protein (CP) which encapsidates the viral genome through helical arrangement of its multiple subunits [4].* Hc-Pro* is a multifunctional protein and a proteinase, which is responsible for the multiplication of virus genome, systemic virus movement [5], and suppression of plant RNA silencing machinery [6] by interacting with numerous host factors.

A conserved DAG (Aspartic acid-Alanine-Glycine) motif of* CP* is responsible for aphid mediated transmission of virus in combination with* Hc-Pro* which has a conserved KITC (Lycine-Isoleucine-Threonine-Cysteine) motif that binds in aphid stylet [7]. There are two hypotheses about aphid mediated* Potyvirus* transmission, including the bridge hypothesis and the direct hypothesis. The bridge hypothesis reported that the N-terminal region of* Hc-Pro* (KITC motif) recognizes an unknown receptor in the aphid stylets. At the same time or subsequently, an* Hc-Pro* downstream motif, presumably containing the PTK motif, undergoes a specific interaction with the DAG motif on the virus* CP*, thereby mediating retention of the virions at appropriate sites in the vector [8].

Over the last few decades, considerable efforts have been made to control SCMV using diverse approaches. Pathogen derived resistance through coat protein mediated resistance has been used extensively to develop resistance against different virus groups. It has been reported that sense SCMV* CP* gene construct transformed into sugarcane showed different level of resistance when challenged with SCMV [9]. Furthermore, multiple sugarcane lines were also generated through the transformation of the coding sequence of* CP* gene of* Sorghum mosaic virus* (SrMV) strain H into sugarcane. However, some of these sugarcane transgenic lines displayed the mosaic symptoms after being challenged with SCMV-H in field trials [10].

RNA silencing is an evolutionarily conserved gene regulation mechanism in eukaryotes, which plays a fundamental role in controlling both regulation of endogenous gene expression and defense against invasive nucleic acids such as viruses and transposable elements [11, 12]. RNA silencing is induced by double stranded RNA (dsRNA) or hairpin RNA (hpRNA), which is processed into 21–24 nucleotide (nt) small interfering RNA (siRNA) duplex by Dicer or Dicer-like (DCL) protein. One strand of the siRNA duplex is incorporated into the Argonaute protein to form RNA-Induced Silencing Complex (RISC) and guides RISC to single-stranded RNA via sequence complementarity, resulting in Argonaute-mediated cleavage of the target RNA [11, 13].

hpRNA-induced silencing has been established to be a powerful tool to developing plant viral resistance through the silencing of viral RNA. hpRNA targeting SrMV* CP* gene showed approximately 87.5% resistance against SrMV in transgenic sugarcane plants [14]. Evidence about long hpRNA construct targeting multiple genes of* Rice black streaked dwarf virus* was successfully used to regenerate stable and resistant lines against virus in rice [15].

This paper reports the expression analysis of hpRNA, targeting simultaneously* CP* and* Hc-Pro* genes of SCMV, in a model rice plant. The approach was designed to generate SCMV resistance by silencing viral genes that play roles in virus transmission, encapsulation and multiplication, and counter defence against RNA silencing. The validation of the approach not only will lead to the development of SCMV resistant sugarcane but also will provide valuable material to developing resistance in other crops.

## 2. Materials and Methods

### 2.1. Preparation of the Ubi-hpCP:Hc-Pro Construct

**Figure 1:**
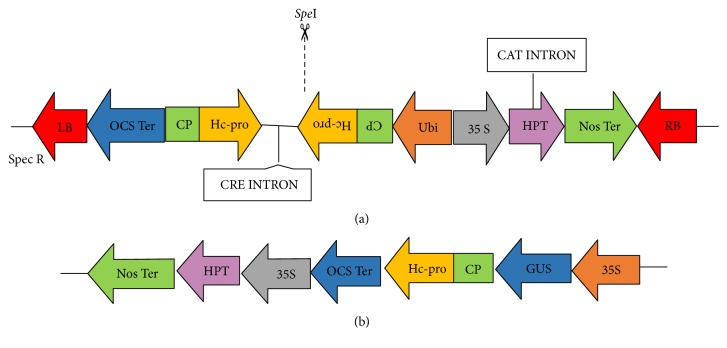
Schematic diagrams representing the chimeric CP:Hc-Pro hairpin RNA construct Ubi-hpCP:Hc-Pro (a) and the target reporter gene construct 35S-GUS:CP:Hc-Pro (b). 35S,* Cauliflower mosaic virus* 35S promoter; Ubi, maize ubiquitin promoter; HPT, hygromycin phosphate transferase gene; OCS Ter,* Agrobacterium* octopine synthase gene terminator sequence; Nos Ter,* Agrobacterium* nopaline synthase gene terminator; CAT INTRON, first intron of castor bean catalase-1 gene; CRE INTRON, the third intron of the* Aegilops tauschii* go35 NBS-LRR (go35) gene;* Spe*I, restriction site used for Southern blot analysis; LB, T-DNA left border; RB, right border.

Consensus sequences of* CP* and* Hc-Pro* genes of SCMV, 240 bp each, were selected and fused into a chimeric fragment for use as the trigger DNA sequence in the hpRNA cassette. These targeted regions were selected by retrieving the multiple sequences of* CP* and* Hc-Pro* genes (about 50 sequences for each gene) from NCBI database and aligned by using ClustalW [16]. A consensus sequence (240 bp) from each gene was selected to form the fusion sequence. This CP:Hc-Pro fusion sequence was synthesized by Gene-Art™ Gene Synthesis (Thermo Fisher Scientific, Waltham, MA) (Figure  S1 in Supplementary Material available online at https://doi.org/10.1155/2017/1646140) and assembled into the hpRNA cassette in both sense and antisense gene orientations. For directional cloning into the pStarling vector, restriction sites of* Kpn*I/*Spe*I and* Bam*HI/*Sma*I were incorporated at 5′ and 3′ ends of fusion fragment, respectively. The expression cassette in pStarling was excised with* Not*I and inserted into pWBVec8 binary plant expression vector [17] (Figure 1(a)). The vector was transformed into* Agrobacterium tumefaciens* AGL1 strain by electroporation method for plant transformation. pWBVec8 binary vector [17] was also transformed as an empty vector control.

### 2.2. Preparation of 35S-GUS: CP-Hc-Pro Fusion Target Construct

The synthesized CP: Hc-Pro fusion fragment was transcriptionally fused with coding sequence of* Escherichia coliβ*-glucuronidase gene (GUS) which was subcloned into pART7 [18] at the* Bam*HI and* Xho*I sites between the CaMV 35S promoter and the octopine synthase (OCS) terminator. The cassette was then excised with* Not*I and cloned into pWBVec2a to form the binary vector suitable for* Agrobacterium*-mediated transformation (Figure 1(b)).

### 2.3. Rice Transformation

The seed of Nipponbare variety of rice* (Oryza sativa)* was selected and cultured on N6D medium for callus induction. Calli were transformed by the Ubi-hpCP:Hc-Pro and empty vector constructs using* Agrobacterium*-mediated transformation according to Hiei et al. [19].

### 2.4. Particle Bombardment of Rice Calli and Transgenic Rice Leaves

Nipponbare rice seed were germinated on N6D (Rice callus induction) medium [19] for 4–6 weeks. Healthy looking rice calli or leaves of transgenic rice were selected for particle bombardment. Plasmid DNA (1 *μ*g) was mixed with gold particles in 0.6 *μ* in diameter, and 2.5 M CaCl_2_ (16 *μ*L) was added, which aids the binding of negatively charged DNA molecules to gold particles. After brief vortexing, 0.1 M spermidine solution (6.4 *μ*L) was added into the mixture for protection from endonucleases. These DNA-coated gold particles were bombarded into rice calli or leaves using PDS 1000-He Biolistic particle delivery system at 1100 Psi pressure and 26 inch Hg vacuum at 6 cm optimized target distance. GUS expression was analyzed using histochemical staining 2-3 days after bombardment.

### 2.5. Histochemical GUS Staining

GUS staining of rice calli and leaves was carried out using 5-bromo-4-chloro-3-indolyl glucuronide (X-Gluc) solution (0.1 M NaPO_4_, 0.1% Triton X-100, 0.5 mM potassium ferrocyanide, 0.5 mM potassium ferricyanide, 2 mM X-Gluc, and 10 mM EDTA pH 7.0) according to Jefferson et al. [20]. Bombarded calli and leaves were dipped in X-Gluc solution and incubated for 3-4 hrs at 37°C. The experiment was repeated three times.

### 2.6. RNA Extraction and Northern Blot Analysis

Total RNA was extracted from leaf of transgenic rice plants using TRIzol® Reagent (Thermo Fisher Scientific) following the manufacturer's recommendation.

For Northern blot analysis of target gene expression, total RNA (15 *μ*g) was separated in 1.3% formaldehyde-agarose gel and blotted onto HyBond-N nylon membrane (GE Healthcare Amersham, Rydalmere, NSW, Australia). ^32^P-labelled antisense CP:Hc-Pro RNA probe was prepared by linearizing the CP:Hc-Pro plasmid in the pENTR™/D-TOPO® vector with* Spe*I, followed by in vitro transcription using Sp6 RNA polymerase in the presence of ^32^P-UTP. Hybridization was performed as previously described [21].

For small interfering RNA (siRNA) detection, approximately 30 *μ*g total RNA was separated in 17% denaturing polyacrylamide gel. RNA was electroblotted to HyBond-N^+^ nylon membrane (GE Healthcare Amersham) using 0.5x TBE buffer. After UV cross-linking, the membrane was hybridized at 42°C with ^32^P labeled antisense CP:Hc-Pro RNA probe [22]. Washing of hybridized membrane was carried out for three times at 42°C with 2x SSC containing 0.2% SDS. Hybridized membranes were visualized and recorded with phosphorimager (FLA-5000, Fuji Photo Film, Tokyo, Japan) after overnight exposure. The intensity of the 21–24 nt siRNA bands and the corresponding U6 RNA bands was determined three times against the background of the gel blots using the Multi Gauge Version 3.0 software (FUJIFILM). The average siRNA band intensity was then normalized against the average U6 RNA band intensity to generate the quantification data.

### 2.7. DNA Extraction, PCR Detection, and Southern Blot Analysis

Genomic DNA of rice plants was extracted from leaf tissue using CTAB protocol according to [23]. Forward (5′-ATCTCACCGACTACAGCTTAG-3′) and reverse (5′-GGTGTTACGTGTTTTTCATATGC-3′) primers were used for PCR detection of the Ubi-hpRNA transgene using the following conditions: initial denaturation at 95°C for 5 min, followed by 35 cycles of 95°C for 30 s, 54°C for 30 s, and extension at 72°C for 1 min, with a final extension at 72°C for 5 min.

For Southern blot analysis of transgene copy number, approximately 15 *μ*g of RNase A-treated rice genomic DNA was digested with* Spe*I and separated in 1% agarose gel along with 1 kb plus DNA marker (Gene Ruler, Thermo Fisher Scientific, Walthan, MA) in 1x TBE buffer and transferred onto the Nylon-N^+^ membrane (GE Healthcare Amersham). Probe was prepared by excising the CP:Hc-Pro fragment from the TOPO vector with* Spe*I and* Xho*I followed by gel purification. Purified fragment was labeled with ^32^P radioactive dCTP using Deca label™ DNA labeling kit (Thermo Fisher Scientific) and hybridized with membrane at 65°C in hybridization buffer as previously described [24]. After hybridization, membranes were washed in 1x SSC for 20 min first at room temperature, followed by washes at 50°C and 60–65°C. Blots were visualized and recorded by FLA-5000 phosphorimager (Fuji Photo Film) after overnight exposure.

### 2.8. Assay of Transgenic Rice Plants for SCMV Resistance

Transgenic rice plants at 5–10 leaf stage were inoculated with SCMV-strain A by rubbing carborundum-dusted leaves with extract of SCMV-infected sugarcane leaves in 0.1 M sodium phosphate buffer (pH 7.0). Infected leaves were harvested at 25 days after inoculation (DPI), and total RNA was extracted using TRIzol reagent (Thermo Fisher Scientific). The presence of SCMV was analyzed using RT-qPCR using a pair of* CP* and* Hc-Pro* specific primers**:** 5′-TTACAACGAAGATGTTTTCC-3′ (CP-F), 5′-CTGAAATAGTAAATACGAGG-3′ (CP-R) and 5′-CACAGAGCACACACCTACC-3′ (Hc-Pro-F), 5′-CCCAAATTCATCATCCGATAG-3′ (Hc-Pro-R). The rice *β*-tubulin gene (GenBank Accession number # XM_015794238) was used as the internal reference for the RT-qPCR, using the following PCR primers: TUB-F 5′-GCTGACCACACCTAGCTTTG-3′ and TUB-R 5′-AGGGAACCTTAGGCAGCATG-3′. RT-qPCR was performed in three technical replicates in the Rotor-Gene Real-Time PCR System (Qiagen, Hilden, Germany) using Platinum* Taq* polymerase (Invitrogen, Thermo Fisher Scientific) and SYBR green.

## 3. Results

### 3.1. Development of Ubi-hpCP:Hc-Pro and 35S-GUS:CP:Hc-Pro Constructs

Selection of a suitable target gene sequence can be important for successful knockdown by RNA interference-based approaches. The hpRNA construct was developed targeting* CP* and* Hc-Pro* genes to interfere with the encapsidation, vector mediated virus transmission, and RNA silencing suppressor simultaneously. The consensus sequences of 240 bp each of* CP* gene sequences (between 602 and 841 nt) covering the conserved DAG motif near N-terminus and* Hc-Pro* gene sequence (between 121 and 359 nt) containing the N-terminus KITC motif were selected through multiple sequence alignments (data not shown). The selected* CP* and* Hc-Pro* gene sequences were subsequently joined together to form a chimera sequence (CP:Hc-Pro) which was arranged in an inverted repeat configuration in the hpRNA construct and successfully cloned under the maize ubiquitin promoter (Ubi), to develop Ubi-hpCP:Hc-Pro expression construct (Figure 1(a)). The chimera sequence was fused transcriptionally with GUS reporter gene and subcloned into pART7 [18] between CaMV 35S promoter and the octopine synthase (OCS) terminator, to develop the 35S-GUS:CP:Hc-Pro target construct for detecting the hpRNA silencing efficiency (Figure 1(b)).

### 3.2. The Ubi-hpCP:Hc-Pro Construct Induces Efficient Target Gene Silencing in Rice Callus Tissue

**Figure 2:**
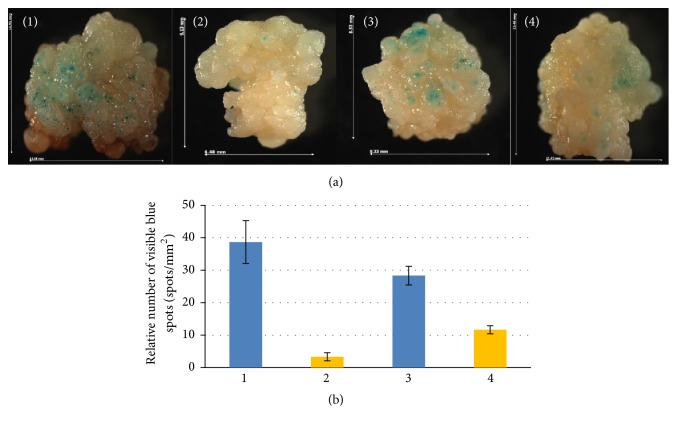
Transient expression assay to evaluate Ubi-hpCP:Hc-Pro construct in rice callus tissue. (a) X-glucuronide staining of rice callus tissue bombarded with (1) 35S-GUS:CP:Hc-Pro, (2) 35S-GUS:CP:Hc-Pro + Ubi-hpCP:Hc-Pro, (3) 35S-GUS:Y-Sat, and (4) 35S-GUS:Y-Sat + Ubi-hpCP:Hc-Pro constructs. Scale bar shows length and width of callus. (b) Histogram showing the average number of blue spots per square millimeter of bombarded rice callus tissue. Error bar represents standard deviation (*n* = 3).

The efficacy of the Ubi-hpCP:Hc-Pro expression construct was validated through a transient expression assay by biolistic delivery of both the targeted GUS reporter gene construct (35S-GUS:CP:Hc-Pro) and the Ubi-hpCP:Hc-Pro construct into rice callus tissue. Another GUS construct (35S-GUS:Y-Sat), with a 3′ fusion of the* Cucumber mosaic virus* Y-satellite RNA sequence [25], was included as a control target. Histochemical staining of the bombarded rice calli showed a clear reduction of blue spots that represented GUS expressing cells, upon cobombardment with the Ubi-hpCP:Hc-Pro construct (Figure 2(a)). On average, 38 blue spots per square millimetre area were observed in rice calli bombarded with 35S-GUS:CP:Hc-Pro alone, and this was in contrast to only about 3 blue spots in calli cobombarded with 35S-GUS:CP:Hc-Pro and Ubi-hpCP:Hc-Pro (Figure 2(b)). This result indicated that the Ubi-hpCP:Hc-Pro construct, comprising the* CP* and* Hc-Pro* sequences, induced sequence-specific suppression of gene expression against the CP:Hc-Pro sequence in the 35S-GUS:CP:Hc-Pro target construct, resulting in downregulation of GUS expression and hence diminished number of blue spots. It is worth noting that cobombardment with the Ubi-hpCP:Hc-Pro construct also reduced the number of blue spots from the expression of the 35S-GUS:Y-Sat construct, but at a lesser degree from an average of 27 per square millimetre to 12. It is unclear why this reduction occurred but it could be due to the sharing of the OCS terminator sequence between the hpRNA and the 35S-GUS:Y-Sat constructs that could induce transgene cosuppression.

### 3.3. Transgenic Rice Plants Containing Ubi-hpCP:Hc-Pro Accumulate siRNAs

**Figure 3:**
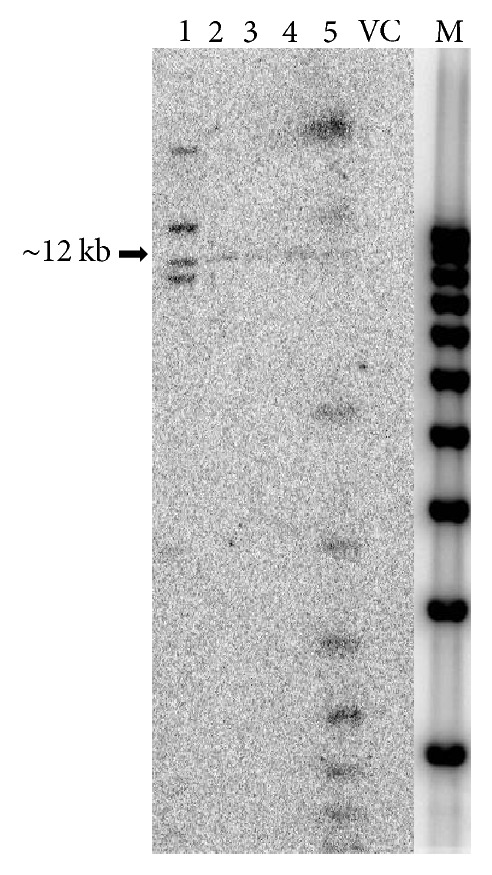
Southern blot analysis of T_0_ transgenic rice plants. DNA extracted from transgenic rice leaves was digested with* Spe*I restriction enzyme, separated in 0.8% agarose gel, transferred to HyBond-N^+^ Nylon membrane, and hybridized with radioactive labeled CP:Hc-Pro DNA probe. Numbers 1–5 indicate five Ubi-hpCP:Hc-Pro transgenic lines. VC, transgenic rice plants containing the empty vector control transgene. M, DNA marker (1 kb plus Gene Ruler). The arrow indicates the single transgene band in lines 2, 3, and 4.

The rice plant, a model monocotyledonous species, was used as a surrogate of sugarcane because of its transformation efficiency, compared to the other cereal crops, such as wheat, maize, and barley and the conservation of gene sequences [26–30]. The Ubi-hpCP:Hc-Pro and the empty vector control (VC) constructs were transformed into rice using* Agrobacterium*-mediated methods, generating the six independent transgenic lines, respectively. One empty vector control and five Ubi-hpCP:Hc-Pro primary transgenic lines (T_0_) were analyzed for transgene copy number by Southern blot hybridization and for the accumulation of siRNAs by Northern blot hybridization. Empty vector control line showed no CP:Hc-Pro hybridization signals on Southern blot while lines #2, #3, and #4 showed a hybridizing band indicating that each of these lines may contain a single copy of transgene. The size of the hybridizing band for lines #2, #3, and #4 appears to be the same (~12 kb), which seems to indicate that they came from a single transgenic event. This is despite the fact that plants were derived from different callus lines. Lines #1 and #5 contained multiple copies as indicated by the multiple hybridizing bands on the Southern blot (Figure 3).

**Figure 4:**
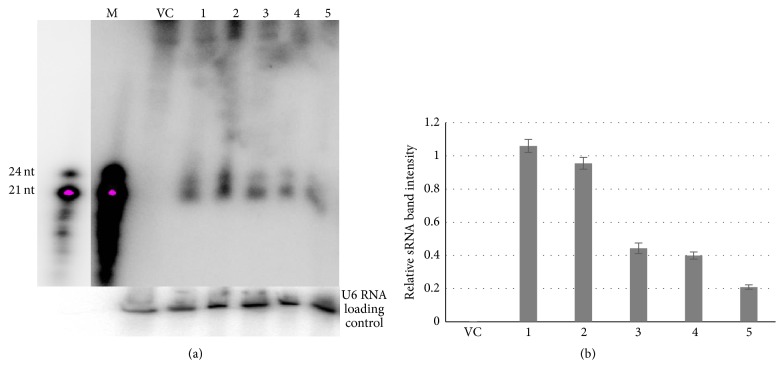
T_0_ transgenic rice plants express 21–24 nt siRNAs. (a) Northern blot hybridization for the detection of siRNA from transgenic rice lines. Twenty-five *μ*g of total RNA extracted from leaves of transgenic plants was hybridized with antisense CP:Hc-Pro RNA probe (the upper panel). The U6 small nuclear RNA was hybridized as a loading control (the lower panel). Lane M represents the 21–24 nt radiolabeled RNA size marker, with a less-exposed picture given on the left to clearly identify the 21 and 24 nt bands. VC is transgenic rice plants containing the empty vector control transgene while, Lanes 1–5 are the same five Ubi-hpCP:Hc-Pro transgenic rice lines shown in Figure 3. (b) Histogram representing the relative intensity of the sRNA band on the Northern blot above.

Small interfering RNAs (siRNAs) 21–24 nts in size, corresponding to the CP:Hc-Pro sequence of the Ubi-hpCP:Hc-Pro, were readily detected by Northern blot analysis in all the five independent lines (Figure 4(a)), indicating that the Ubi-hpCP:Hc-Pro was expressed and the resulting hpRNA processed by Dicer (DCLs) enzyme which recognizes and cleaves the dsRNA into siRNAs. The size distribution of the siRNA was consistent with that of hpRNA-derived siRNAs in* Arabidopsis (Arabidopsis thaliana)*, consisting of 21, 22, and 24 nt size classes [31], (although the 21 and 22 nt species were not clearly separated) (Figure 4(a)). This indicates that the CP:Hc-Pro hpRNA was processed in transgenic rice by DCL4, DCL2, and DCL3, respectively, corresponding to the pattern observed in* Arabidopsis* [32]. In a previous report, DCL4 dependent 21 nt siRNAs were the predominant species of siRNAs derived from the SCMV [33]. Moreover, DCL2 and DCL4 function redundantly in maize and* Arabidopsis* plants to produce higher level of 21 nt and 22 nt siRNAs [33, 34]. It is noteworthy that in the different transgenic lines the siRNA hybridization signals showed different intensity, indicating a variable level of siRNA in these lines (Figure 4(b)). Interestingly, the high-copy-number transgenic line #5 did not accumulate more siRNAs; instead, it appears to generate slightly less siRNAs, suggesting that the multiple copy transgene insertion may have resulted in transcription repression of the hpRNA transgene [35].

### 3.4. The Ubi-hpCP:Hc-Pro Transgene Confers Silencing to a Biolistically Delivered Target Gene

The efficacy of Ubi-hpCP:Hc-Pro transgene against the target viral sequences was shown in a transient expression assay by delivering the 35S-GUS:CP:Hc-Pro target construct into transgenic rice leaves using particle bombardment. Strong GUS expression was observed in leaf tissue of the empty vector control transgene but not in the leaves of the Ubi-hpCP:Hc-Pro transgenic plants (three replicates of all transgenic lines), indicating that siRNAs derived from the Ubi-hpCP:Hc-Pro induced silencing against the 35S-GUS:CP:Hc-Pro target construct by targeting CP:Hc-Pro fusion sequence (Figure  S2).

### 3.5. Ubi-hpCP:Hc-Pro Transgene Stably Inherited in T_1_ Generation and Confers Resistance against SCMV

**Figure 5:**
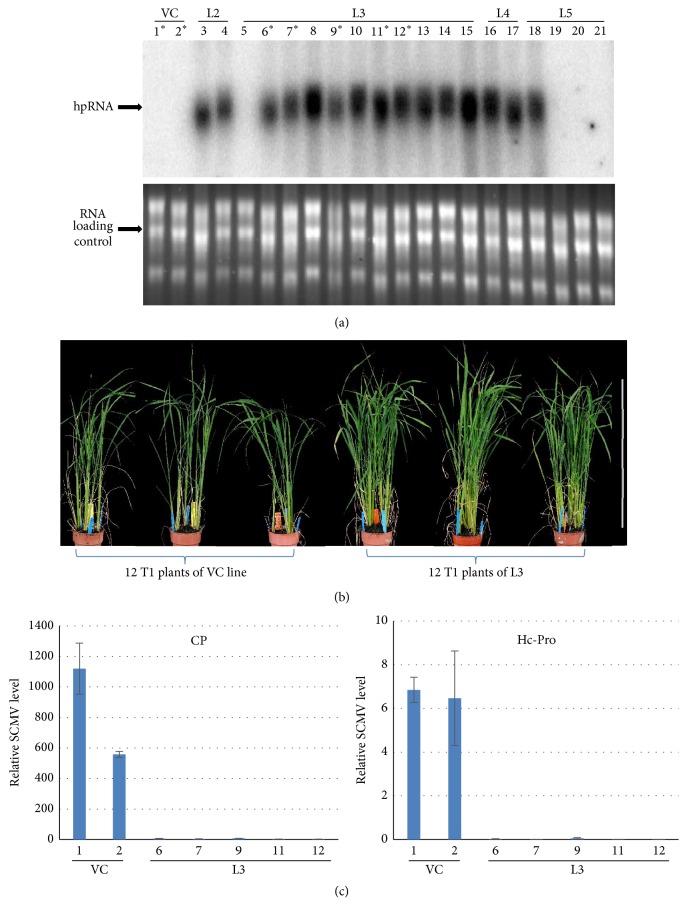
Ubi-hpCP:Hc-Pro transgene expression and SCMV resistance analysis in T_1_ generation. (a) Northern blot hybridization of T_1_ progeny of four independent Ubi-hpCP:Hc-Pro lines (#2, #3, #4, and #5) and an empty vector control line (VC) using ^32^P-labelled antisense CP:Hc-Pro RNA as probe, which shows Ubi-hpCP:Hc-Pro expression from #2, #3 (except for one plant), #4, and one of #5 plants, but not from the vector control and the other three #5 plants. Lower panel shows RNA loading control. Asterisks indicate plants analysed in (c). (b) Vector control and Ubi-hpCP:Hc-Pro transgene T_1_ transgenic rice plants inoculated six weeks before with SCMV. (c) Quantitative real-time RT-PCR analysis of SCMV accumulation in infected VC and hpRNA transgenic T1 plants using* CP* (left) or* Hc-Pro* (right) primers.

To investigate if the Ubi-hpCP:Hc-Pro transgenic plants were resistant to SCMV infection, seeds were collected from lines #2, #3, #4, and #5 primary transgenic plants (line #1 did not produce seeds) from which T_1_ plants were established and assayed for transgene expression and SCMV resistance. The Ubi-hpCP:Hc-Pro transgene expression, as indicated by the hybridizing signals of the unprocessed CP:Hc-Pro hpRNA on the Northern blot, was clearly detected in the T_1_ progeny of the single-copy lines #2, #3, and #4, indicating stable inheritance from the primary transgenic plants (Figure 5(a)). However, for line #5, the highest-copy-number transgenic line, three of the four T_1_ plants analyzed showed no Ubi-hpCP:Hc-Pro signal, which may indicate the transcriptional suppression of the Ubi-hpCP:Hc-Pro transgene in these individuals of high copy number transgene insertion. It is also noticeable that plant #5 from line 3 also showed no Ubi-hpCP:Hc-Pro hybridization signals (Figure 5(a)).

To examine if the Ubi-hpCP:Hc-Pro transgene could confer resistance to SCMV infection, a number of T_1_ plants of line #3, shown to express high levels of Ubi-hpRNA in the Northern blot (Figure 5(a)), together with T_1_ plants of an empty vector control line, were inoculated with SCMV. The 12 T_1_ Ubi-hpCP:Hc-Pro plants showed visibly higher growth vigor than the 12 empty vector control plants at 6 weeks after inoculation, implying resistance to SCMV (strain A, a mild strain of SCMV) infection (Figure 5(b)). SCMV strain A infection studies typically showed stunted growth with mild mosaic pattern without necrosis in its natural hosts that are sugarcane, maize, and sorghum [36]. In the present study, no clear mosaic symptoms were observed in the SCMV-inoculated plants possibly because rice is not the natural host of SCMV and very mild mosaic symptoms were reported through artificial inoculation of SCMV [36]. To confirm the successful SCMV resistance, RNA was extracted from a subset of the inoculated plants and analyzed for the presence of SCMV using RT-qPCR with* CP* and* Hc-Pro* specific primers. The empty vector control plants showed high levels of SCMV* CP* and* Hc-Pro* RNA (Figure 5(c)). In contrast, no significant amount of SCMV RNA was detectable in all five T_1_ transgenic plants analyzed, indicating that the Ubi-hpCP:Hc-Pro transgenic plants (from line #3) were highly resistant to SCMV infection. Both the* CP* and* Hc-Pro* target sequences were similarly silenced in the SCMV-infected Ubi-hpCP:Hc-Pro transgenic plants (Figure 5(c)), indicating that the transgene was effective against both genes.

## 4. Discussion

SCMV is an agronomically important* Potyvirus* infecting a variety of monocotyledonous species including important crop plants such as maize and sugarcane. It is transmitted in nature by five different insect vectors:* Aphis craccivora*,* Aphis gossypii*,* Macrosiphum euphorbiae*,* Rhopalosiphum maidis*, and* Rhopalosiphum padi* [37]. Outbreak of this virus often causes severe damage to crops leading to high cost to crop yields in more than 70 countries [38]. According to the survey conducted between 2008 and 2010 in Pakistan, the highest incidence of virus infection in sugarcane fields reached up to 38% in the Punjab province of Pakistan during the year 2010 [39].

Because of the potential damage SCMV could do to the agronomically important monocotyledonous crops, many efforts have been made to control the spread and multiplication of this virus. It was reported that protection against severe SCMV strains was achieved in a number of different crops by inoculating plants with mild virus strain through the mechanism of viral cross protection [40, 41]. Attempts have also been made in the selection and breeding for naturally occurring resistant crop varieties for controlling SCMV. For example, a European maize inbred line was found to be completely resistant to SCMV and Maize dwarf mosaic virus in green house and field tests [42]. As a number of plant viruses such closteroviruses, comoviruses, tymoviruses, and nepoviruses utilize cysteine proteinases in their polyprotein processing, a cysteine proteinase inhibitor, cystatin, has been transgenically expressed in tobacco to control the multiplication of these viruses [43], which could be potentially used to control SCMV. However, an effective method for controlling SCMV and SCMV-caused diseases has so far not been found.

RNA silencing-based approaches, especially the long hpRNA technology, has been successfully used to engineer plant resistance against viruses, including agronomically important potyviruses, such as Wheat streak mosaic virus, Potato virus Y, and Plum pox virus [44, 45]. Various regions of the potyviral genome, such as those encoding the* NI*a protease and* Hc-Pro*, have been selected as the target sequences for the hpRNA transgenes in such experiments. For the SCMV, attempts were also made to transform maize with an hpRNA construct targeting the* CP* gene, generating transgenic plants with variable levels of SCMV resistance [46]. Similarly, SrMV resistant sugarcane transgenic lines were generated using hpRNA transgene targeting* CP* region showing different levels of virus resistance [47]. Thus, hpRNA transgenes provide a potential opportunity for developing SCMV resistance in important crops such as sugarcane.

While targeting a single viral gene by hpRNA transgenes has proved to be successful, we chose a strategy of simultaneously targeting two essential SCMV genes,* CP* and* Hc-Pro*. While* CP* is essential for viral encapsidation and transmission,* Hc-Pro* has multiple functions including the function of countering RNA silencing. It was anticipated that simultaneous silencing of the* CP* and the* Hc-Pro* genes would both cause direct downregulation of viral RNAs and enhance host antiviral silencing activity, therefore resulting in robust virus resistance in the transgenic plants. We selected 240 bp segments from each of the* CP* and* Hc-Pro* genes that correspond to conserved functional motifs, with the purpose of achieving broad resistance against the various SCMV virus isolates. In addition, the maize ubiquitin promoter was chosen to drive the expression of the Ubi-hpCP:Hc-Pro transgene, which has been shown to be suitable for high levels of transgene expression in rice and other monocot species [48]. Finally, in the Ubi-hpCP:Hc-Pro construct the sense and antisense CP:Hc-Pro sequences are separated by a spliceable intron, which is suggested to enhance the efficacy of hpRNA transgenes [49].

The efficacy of the Ubi-hpCP:Hc-Pro transgene was first demonstrated by showing that it could induce efficient silencing against the 35S-GUS:CP:Hc-Pro fusion transgene when transiently coexpressed in rice callus tissue using particle bombardment. When stably transformed into rice plants, this Ubi-hpCP:Hc-Pro transgene gave rise to siRNAs across the independent transgenic lines, which conferred silencing against the biolistically delivered 35S-GUS:CP:Hc-Pro fusion transgene. The size distribution of the siRNAs is consistent with the typical pattern of hpRNA-derived siRNAs [31], with the 21-22 nt siRNAs being more abundant than the 24 nt species. Interestingly, the abundance of siRNAs showed no positive correlation with the copy number of Ubi-hpCP:Hc-Pro transgene insertion, and it appeared that the higher the copy number is, the less the siRNA accumulates. Analysis of Ubi-hpCP:Hc-Pro transcript accumulation in the subsequent T_1_ population further suggested that the Ubi-hpCP:Hc-Pro transgene is subject to transcriptional silencing when the number of transgene insertions is very high, which is consistent with previous reports [50]. It was previously reported that high copy number or polyploid progenitor line undergoes partial or complete transgene silencing which is caused by transacting silencing of active and inactive epialleles [51]. This suggests that for achieving stable virus-resistance, transgenic plants with a simple transgene insertion pattern should be selected.

Virus infection assays of the single-copy transgenic line expressing high levels of the Ubi-hpCP:Hc-Pro showed that the transgenic plants are highly resistant to SCMV, which displayed a more vigorous growth than vector control transgenic plants, with almost no detectable SCMV RNA in contrast to SCMV RNA in the vector control plants.

In conclusion, we demonstrate here that high-level SCMV resistance can be achieved using an hpRNA transgene targeting two essential viral genes at the same time. While this strategy has not been tested in sugarcane it is anticipated that the Ubi-hpCP:Hc-Pro construct simultaneously targeting the downregulation of both* CP* and* Hc-Pro* expression in SCMV will confer SCMV resistance when transformed into sugarcane.

## Supplementary Material

Figure S1: Sequence of coat protein (*CP*) (In Red) and Helper component proteinase (*Hc-Pro*) (In Blue) containing the conserved motifs of both genes fused together. Multiple unique restriction sites were added in the start and end of fused sequence. Respective sequence was synthesized from GeneArt™ Gene Synethesis for further cloning into Hairpin vector.
Figure S2: GUS expression assay in the Ubi-hpCP:Hc-Pro transgenic rice leaves. In the representative images, leaves of the rice plants transformed with the empty vector control (A, B) and those transformed with Ubi-hpCP:Hc-Pro (line #3) (C) were bombarded with 35S-GUS:CP: Hc-Pro fusion construct, and stained with X-Gluc. Prominent GUS expression (blue spots) was detected in the empty vector control leaves but GUS expression was hardly discernable in the leaves derived from rice plants transformed with Ubi-hpCP:Hc-Pro, indicating that GUS silencing was induced by the Ubi-hpCP:Hc-Pro transgene. Scale bar = 2 mm. The experiment was repeated for three times.
