# Supplementary material for: Expression Analysis of Hairpin RNA Carrying Sugarcane mosaic virus (SCMV) Derived Sequences and Transgenic Resistance Development in a Model Rice Plant
Source: Biomed Res Int. 2017 Feb 1;2017:1646140. doi: 10.1155/2017/1646140 (PMC5309402; doi:10.1155/2017/1646140)
Supplement: Supplementary file 1 — Figure S1: Sequence of coat protein (CP) (In Red) and Helper component proteinase (Hc-Pro) (In Blue) containing the conserved motifs of both genes fused together. Multiple unique restriction sites were added in the start and end of fused sequence. Respective sequence was synthesized from GeneArt™ Gene Synethesis for further cloning into Hairpin vector. Figure S2: GUS expression assay in the Ubi-hpCP:Hc-Pro transgenic rice leaves. In the representative images, leaves of the rice plants transformed with the empty vector control (A, B) and those transformed with Ubi-hpCP:Hc-Pro (line #3) (C) were bombarded with 35S-GUS:CP: Hc-Pro fusion construct, and stained with X-Gluc. Prominent GUS expression (blue spots) was detected in the empty vector control leaves but GUS expression was hardly discernable in the leaves derived from rice plants transformed with Ubi-hpCP:Hc-Pro, indicating that GUS silencing was induced by the Ubi-hpCP:Hc-Pro transgene. Scale bar = 2 mm. The experiment was repeated for three times. [file 1646140.f1.pdf]

Supplementary data:

***Kpn*I *Xho*I *Bam*HI** **D A G**

CGGggtacc TCGAg gatcc AGTTTGATAGATGGTAT**GATGCCGGT**AAGAAGGAATACGAAAT
TGATGACACACAAATGACAGTTGTCATGAGTGGTCCTATGGTATGGTGCATTGAAAATGGT
AGCTCACCAAACATAAACGGAAATTGGACAATGATGGATGGAGATGAACAAAGGGTTTTT
CCACTCAAACCGGTCATTGAAAATGCATCTCCAACTTTCCGACAAATTATGCATCATTTTA
GTGATGCAGCTGAAGAGAATGGCATTGTTAGAAATACTATTTCACTC

K I T C

AACTTTT**AAAATCACATGC**AAAACGTGTAATATTGACGATCTGGAATTATCAGATGATGAA
TTTGGGGCCAAGTTATATAGCAATCTGCAGCGTATTGAAGAAAAGCAACGTGAATATCTTG
CTAAAGATCAAAAACTTCTACGCATGATACACTTCGTAAAGGATCGGTGTAACCCAAAATT
TTCACATTTGCCTCTACTATGGCAA**cccggg AAGCTT actagt GGTACC CGC**

***Sma*I *Hin*dIII *Spe*I *Kpn*I**

Figure S1: Sequence of coat protein (*CP*) (In Red) and Helper component proteinase (*Hc-Pro*) (In Blue) containing the conserved motifs of both genes fused together. Multiple unique restriction sites were added in the start and end of fused sequence. Respective sequence was synthesized from GeneArt™ Gene Synthesis for further cloning into Hairpin vector.

A **B** **C**

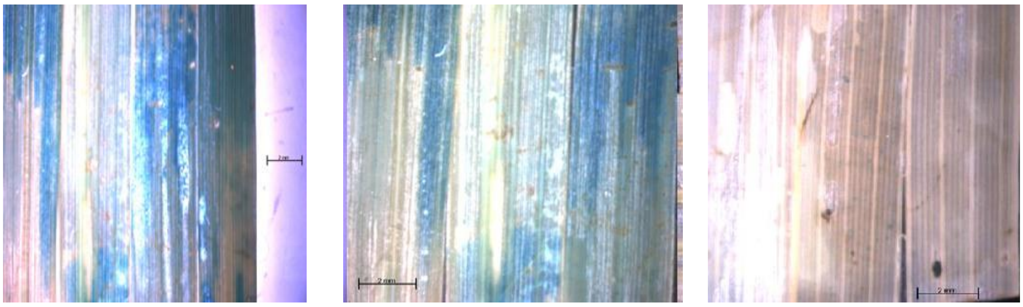

Figure S2: GUS expression assay in the Ubi-hpCP:Hc-Pro transgenic rice leaves. In the representative images, leaves of the rice plants transformed with the empty vector control **(A, B)** and those transformed with Ubi-hpCP:Hc-Pro (line #3) **(C)** were bombarded with 35S-GUS:CP: Hc-Pro fusion construct, and stained with X-Gluc. Prominent GUS expression (blue spots) was detected in the empty vector control leaves but GUS expression was hardly discernable in the leaves derived from rice plants transformed with Ubi-hpCP:Hc-Pro, indicating that GUS silencing was induced by the Ubi-hpCP:Hc-Pro transgene. Scale bar = 2 mm. The experiment was repeated for three times.
